# Malleability of the Folding Mechanism of the Outer Membrane Protein PagP: Parallel Pathways and the Effect of Membrane Elasticity

**DOI:** 10.1016/j.jmb.2011.12.039

**Published:** 2012-02-24

**Authors:** Gerard H.M. Huysmans, Sheena E. Radford, Stephen A. Baldwin, David J. Brockwell

**Affiliations:** 1 Astbury Centre for Structural Molecular Biology, University of Leeds, Leeds LS2 9JT, UK; 2 Institute of Membrane and Systems Biology, University of Leeds, Leeds LS2 9JT, UK; 3 Institute of Molecular and Cellular Biology, University of Leeds, Leeds LS2 9JT, UK

**Keywords:** LPR, lipid-to-protein ratio, *p*NPP, *para*-nitrophenylpalmitate, *di*C_12:0_PC, 1,2-dilauroyl-*sn*-glycero-3-phosphocholine, *di*C_12:0_PE, 1,2-dilauroyl-*sn*-glycero-3-phosphoethanolamine, *di*C_12:0_PS, 1,2-dilauroyl-*sn*-glycero-3-phosphoserine, PTI, Photon Technology International, membrane protein folding, PagP, outer membrane protein, β-barrel, kinetics

## Abstract

Understanding the interactions between membrane proteins and the lipid bilayer is key to increasing our ability to predict and tailor the folding mechanism, structure and stability of membrane proteins. Here, we have investigated the effects of changing the membrane composition and the relative concentrations of protein and lipid on the folding mechanism of the bacterial outer membrane protein PagP. The folding pathway, monitored by tryptophan fluorescence, was found to be characterized by a burst phase, representing PagP adsorption to the liposome surface, followed by a time course that reflects the folding and insertion of the protein into the membrane. In 1,2-dilauroyl-*sn*-glycero-3-phosphocholine (*di*C_12:0_PC) liposomes, the post-adsorption time course fits well to a single exponential at high lipid-to-protein ratios (LPRs), but at low LPRs, a second exponential phase with a slower folding rate constant is observed. Interrupted refolding assays demonstrated that the two exponential phases reflect the presence of parallel folding pathways. Partitioning between these pathways was found to be modulated by the elastic properties of the membrane. Folding into mixed 1,2-dilauroyl-*sn*-glycero-3-phosphoethanolamine:*di*C_12:0_PC liposomes resulted in a decrease in PagP adsorption to the liposomes and a switch to the slower folding pathway. By contrast, inclusion of 1,2-dilauroyl-*sn*-glycero-3-phosphoserine into *di*C_12:0_PC liposomes resulted in a decrease in the folding rate of the fast pathway. The results highlight the effect of lipid composition in tailoring the folding mechanism of a membrane protein, revealing that membrane proteins have access to multiple, competing folding routes to a unique native structure.

## Graphical abstract

**Graphical Abstract:**

**Figure:**
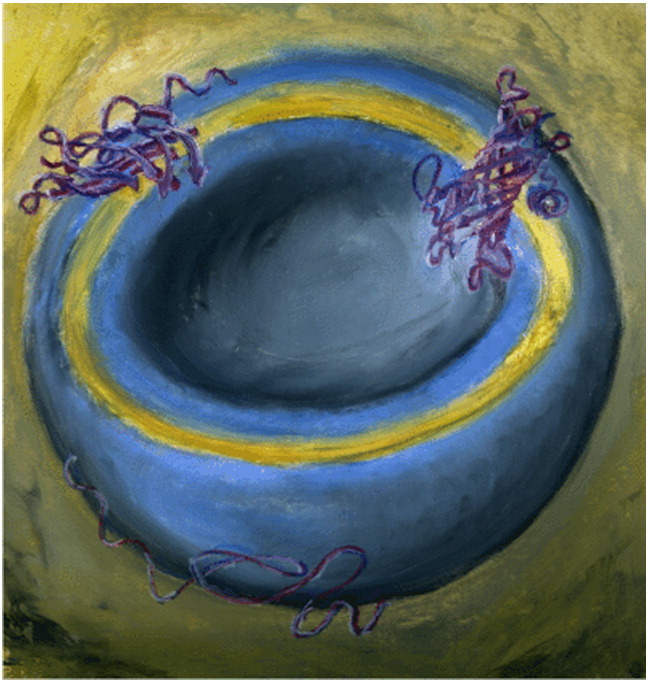

**Research Highlights:**

► Lipid adds complexity when investigating the folding mechanisms of membrane proteins. ► Relative amplitudes of burst and exponential phases are altered by LPR. ► Interrupted refolding assays reveal parallel folding pathways. ► Partitioning between pathways is modulated by chemical composition of the membrane. ► PagP has access to multiple, competing folding routes to the native state.

## Introduction

How the information inherent in the amino acid sequence of a protein enables it to adopt a native, three-dimensional structure remains a fundamental question in structural biology. For soluble proteins, excellent progress towards answering this question has been made by integrating experimental folding studies on small model proteins with computer simulations.^1–10^ By contrast, progress in understanding the folding mechanisms of membrane proteins has been much more limited,^11–14^ in part because of the complexity added to delineating the mechanisms of folding by the membrane environment in which the protein resides. Biological membranes comprise a complex two-dimensional fluid with a heterogeneous lipid composition^15–17^ that largely determines the physicochemical properties of the membrane.^18–21^ The organization of the lipid bilayer (a hydrophobic core flanked by often asymmetrical polar interfaces) poses significant spatial restrictions on the folding process, which are difficult to mimic *in vitro*. In addition, membrane curvature imposes stresses on the bilayer that may be alleviated or exacerbated by protein insertion^22,23^ and hence can also modulate the rate, or efficiency, of folding. In order to understand the native structures of membrane proteins, it is thus necessary to investigate how the lipid membrane contributes to, and/or limits, protein folding, stability and conformational dynamics.

Owing to the hydrophobic nature of the lipid bilayer core, transmembrane segments of integral membrane proteins almost exclusively contain regular secondary structural elements, traversing the membrane either as α-helices or as β-barrels.^24^ The folding mechanism of several β-barrel proteins has been studied in the presence of artificial membranes commencing from a fully or partially denatured state *in vitro*.^25–39^ For the outer membrane β-barrel protein OmpA from *Escherichia coli*, changes in the folding kinetics into bilayers of differing composition were correlated with general membrane properties such as membrane thickness and lateral pressure.^40^ However, the large diversity of folding behaviors observed for a series of β-barrel membrane proteins under comparable conditions also indicates that elucidation of protein-specific interactions with the membrane is likely to be crucial for a full understanding of the protein–membrane relationship.^26^

In this study, we provide a detailed kinetic analysis of the folding mechanism of the outer membrane protein PagP of *E. coli* in a series of physically and chemically diverse bilayers, to delineate how forces present in the lipid bilayer influence the folding process. PagP forms an eight-stranded transmembrane β-barrel that is preceded by an N-terminal α-helix on the periplasmic side of the membrane.^41,42^ Under stress conditions, PagP transfers a palmitoyl-chain from a phospholipid to lipopolysaccharides in the outer leaflet of the outer membrane.^41^ Previously, we have shown that PagP refolds spontaneously with high yield into lipid vesicles of 1,2-dilauroyl-*sn*-glycero-3-phosphocholine (*di*C_12:0_PC) in the presence of 7 M urea^31^ and have presented a detailed analysis of the folding transition state of PagP in a *di*C_12:0_PC bilayer.^30^

Here, we investigated how the folding and unfolding kinetics of PagP are influenced by the protein and lipid concentrations, by the packing of the hydrocarbon core and by the heterogeneity in the membrane–water interface (through variation of the lipid headgroup composition of the bilayer). In all membranes studied, the folding process comprised multiple phases, with a burst phase followed by one or two exponential phases. Combined with interrupted refolding experiments, we reveal that PagP folds to its native, membrane-embedded state by parallel pathways and that the kinetic partitioning between different routes is determined by the elastic bending modulus of the membrane. The results provide new details of how this outer membrane protein folds and provide evidence that the folding of membrane proteins occurs on a complex energy landscape, the properties of which are governed by the lipid-to-protein ratio (LPR), the protein sequence and the nature of the lipid bilayer itself.

## Results

### Folding kinetics of PagP are dependent on protein and lipid concentrations

**Fig. 1:**
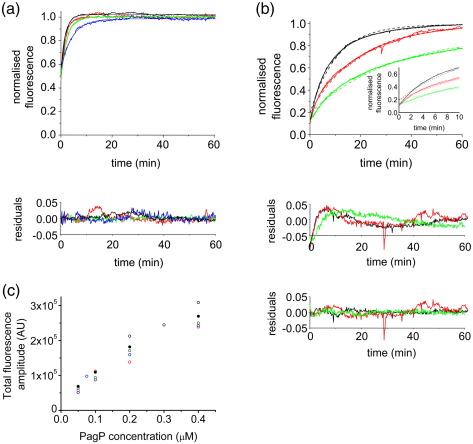
(a) Top: refolding of 0.4 (black), 0.2 (red), 0.1 (green) and 0.05 (blue) μM PagP into *di*C_12:0_PC liposomes at an LPR of 3200:1. Smooth lines through the experimental traces represent fits to a single exponential plus a constant representing the initial burst phase. Bottom: residuals of the fits. (b) Top: refolding of 0.4 (black), 0.3 (red) and 0.2 (green) μM PagP into *di*C_12:0_PC liposomes at an LPR of 400:1. The inset shows an expansion of the first 10 min. Continuous lines through the experimental traces represent fits to a double exponential and a constant representing the burst phase; broken lines represent fits to a single exponential and a constant representing the burst phase. Middle and bottom: residuals to the fits to single and double exponentials, respectively. All experiments were performed in the presence of 7 M urea in 50 mM sodium phosphate buffer, pH 8, at 25 °C. (c) Graph showing that the total amplitude of the folding reaction scales linearly with PagP concentration. This demonstrates that the yield and quality of signal were not affected by folding conditions (LPRs of 400:1, black; 800:1, red; 1600:1, blue; 2400:1, green; 3200:1, filled black).

We have previously shown that 0.4 μM PagP folds into *di*C_12:0_PC liposomes, 100 nm in diameter, in the presence of high concentrations of urea (7–8.8 M) *via* a multistep mechanism that can be studied using tryptophan (Trp) fluorescence.^30,31^ At an LPR of 3200:1, folding is independent of both the protein and the lipid concentrations and, starting from a liposome-adsorbed state, is described by a single exponential at urea concentrations ≥ 7.8 M.^30^ However, between 7 and 7.6 M urea, or when starting from a completely unfolded state, additional kinetic refolding phases are observed.^30^ To investigate the origin of these complexities, we here investigated the refolding kinetics of PagP from a completely unfolded and liposome-dissociated state in the presence of 7 M urea at protein concentrations between 0.05 and 0.4 μM PagP and LPRs between 400:1 and 3200:1. Consistent with previous results,^30^ we showed that the folding kinetics are characterized by a burst phase, attributable to adsorption of PagP to liposomes,^30^ followed by a slower exponential phase (Fig. 1a and b). At high protein concentrations and high LPRs, the latter phase fitted well to a single exponential (Fig. 1a), characterized by a rate constant *k*_f1_, with the most rapid rates obtained at the highest protein concentrations examined (Table 1, column “*k*_f1_”). At the low LPR of 400:1, and in some cases between LPRs of 800:1 and 2400:1, a second exponential with a slower rate constant (*k*_f2_) was required to fit the folding traces satisfactorily (Fig. 1b and Table 1, column “*k*_f2_”). While the differences between the slow and fast rate constants were small (mostly less than 10-fold), these differences were reproducible across replicates and within experiments (fluorescence was monitored on multiple samples using a multi-cell changer) with different refolding kinetic mechanisms observed reproducibly when folding was measured into liposomes under different conditions.

**Fig. 2:**
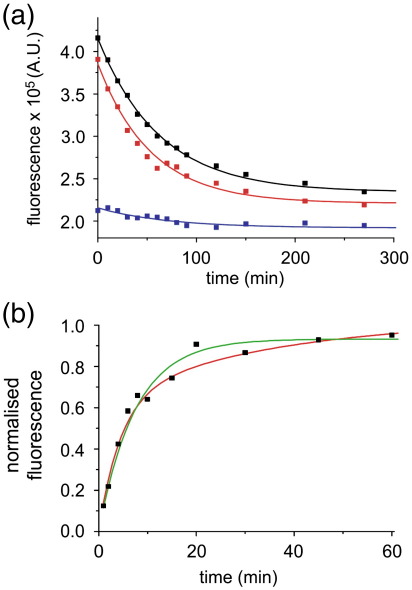
(a) Representative unfolding traces of PagP in 9.6 M urea for samples taken 1 min (blue), 30 min (red) and 60 min (black) after initiation of folding of 0.4 μM PagP into *di*C_12:0_PC liposomes at an LPR of 400:1 in 7 M urea. Lines represent single exponential fits. (b) Time course for the formation of native PagP in *di*C_12:0_PC liposomes (black squares) as measured by the interrupted folding method. The data are fitted to a single (green line) or double (red line) exponential function.

To illustrate trends in the variation of the amplitudes and the rate constants of the observed phases in the folding kinetics with respect to the PagP concentration and LPR more clearly, we expressed the amplitude of the phase(s) following the burst as a percentage of the total amplitude to determine their relative contribution to the folding reaction compared with the amplitude of the burst phase (Table 1, column “% *A*_total_”). The amplitudes of each exponential phase were then expressed as a fraction of this post-burst phase amplitude, to enable their relative contributions to the total kinetic amplitude to be assessed (Table 1, columns “*A*_1_” and “*A*_2_”, see Materials and Methods for details). Data processing in this fashion clearly shows that (1) the burst phase amplitude was reduced significantly (i.e., % *A*_total_ increased,Table 1) at lower LPRs (400–800:1) but did not depend critically on the protein concentration at any LPR measured [note: under all LPRs studied, the amplitude of the reaction scaled linearly with the protein concentration (Fig. 1c)]; (2) the amplitude of the faster exponential phase, associated with *k*_f1_ (*A*_1_), decreased relative to the amplitude of the slower exponential phase, associated with *k*_f2_ (*A*_2_), with decreasing LPR and, in most cases, with decreasing PagP concentration (Table 1); and (3) in agreement with previous results,^30^ PagP folding could be unambiguously described as a unimolecular reaction at LPRs above 1600:1 with PagP concentrations at or above 0.2 μM.

### Complex refolding kinetics of PagP indicate parallel folding pathways

The detection of two exponential phases in the time course of PagP folding at lower LPRs or lower PagP concentrations could reflect the existence of a spectrally detectable intermediate state or stem from attainment of the native state by two distinct folding pathways.^43^ To distinguish between these possibilities, we took samples at different times during folding and then immediately subjected them to unfolding in urea. This was achieved by initiating the folding of 0.4 μM PagP at an LPR of 400:1 in the presence of 7 M urea. Following incubation for defined periods of increasing length (*t*_*i*_), samples were taken, and their unfolding kinetics were measured in the presence of 9.6 M urea (Fig. 2a). If the measured unfolding rate constant equals the expected rate constant for the unfolding of the native state under these conditions, then the amplitude of the unfolding reaction will be proportional to the fraction of native PagP in the refolding mixture at each time point taken.^43^ Apparent unfolding rate constants distinct from that of the native state, by contrast, would be indicative of the formation of a folding intermediate. Unfolding transients of PagP obtained at different times after initiating folding fitted well to single exponentials (Fig. 2a), with a rate constant consistent with the unfolding of native PagP under the applied conditions (0.017 ± 0.001 s^− 1^). Plotting the amplitudes of these *versus* refolding time (Fig. 2b) revealed that formation of the native state occurs in two phases with rate constants of *k*_1_ = 0.23 ± 0.05 min^− 1^ (*A*_1_ = 0.63 ± 0.12) and *k*_2_ = 0.03 ± 0.02 min^− 1^ (*A*_2_ = 0.39 ± 0.09). The resulting rates agree well with the refolding kinetics measured for 0.4 μM PagP at an LPR of 400:1 in the presence of 7 M urea as described above (Table 1). Should these rates result from the formation of sequential states, a delay in the formation of native PagP is expected. The absence of such a delay (Fig. 2b) suggests that the two kinetic phases result from refolding along two (or more) distinct pathways with different rate constants under the conditions employed.

### PagP folds efficiently into lipid bilayers of varying composition

**Fig. 3:**
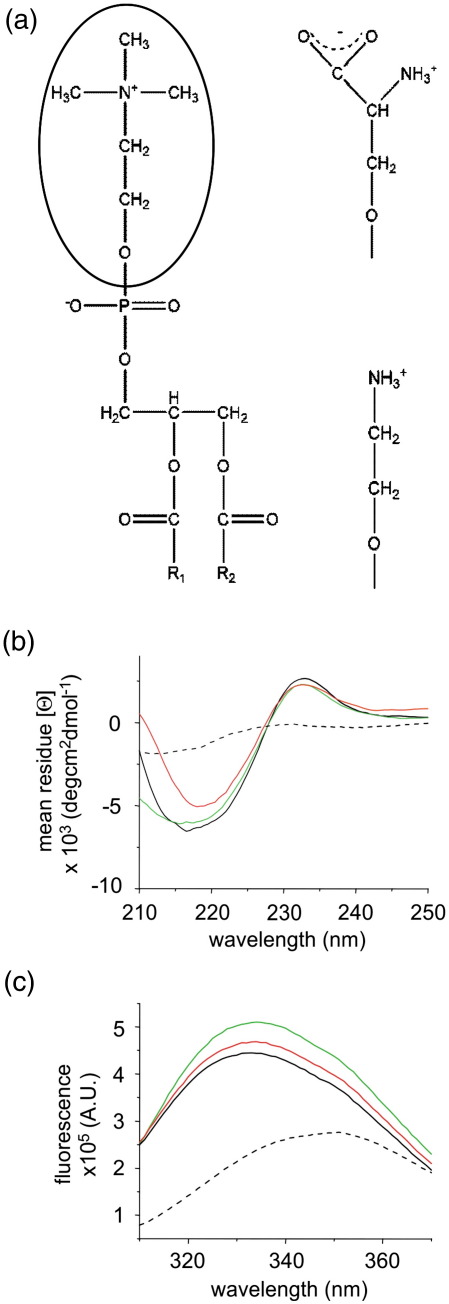
(a) Schematic representation showing the variation in the lipid headgroups used in this study. The choline headgroup (encircled on the left) in *di*C_12:0_PC is replaced by serine and ethanolamine in *di*C_12:0_PS (top right) and *di*C_12:0_PE (bottom right), respectively. (b) CD and (c) tryptophan fluorescence emission spectra of PagP refolded in the presence of 7 M urea in *di*C_12:0_PC liposomes (continuous black line) and in *di*C_12:0_PC liposomes containing 10% w/w *di*C_12:0_PE (red) or 40% w/w *di*C_12:0_PS (green). The unfolded spectrum of PagP in 7 M urea in the absence of liposomes is also shown (dashed black line). PagP concentrations were 5 μM for CD spectroscopy and 0.4 μM for tryptophan fluorescence experiments. All experiments were performed with an LPR of 3200:1 in 50 mM sodium phosphate buffer, pH 8, at 25 °C.

The effects of the hydrophobic thickness of the membrane on the yield and kinetics of folding for various bacterial outer membrane β-barrel proteins have been studied widely.^26,44^ For PagP, such studies reveal that the rates and yields of folding decrease with increasing phospholipid acyl chain length from C_10_ to C_14_.^26^ To further elucidate the influence of the composition of the lipid bilayer on the folding mechanism of PagP, we next investigated the effects of varying the physicochemical properties of the membrane interface on the folding properties of the protein. Changing the composition of the headgroup region *via* incorporation of 1,2-dilauroyl-*sn*-glycero-3-phosphoserine (*di*C_12:0_PS) (up to 40% w/w) or 1,2-dilauroyl-*sn*-glycero-3-phosphoethanolamine (*di*C_12:0_PE) (up to 15% w/w) in *di*C_12:0_PC liposomes allowed modulation of the headgroup charge and volume (Fig. 3a), respectively, and, in the case of the latter, bilayer fluidity.^45–47^

Successful folding of PagP in such membranes was first confirmed using far-UV circular dichroism (CD) and Trp fluorescence emission spectroscopy and by measuring PagP activity towards the substrate analogue *para*-nitrophenylpalmitate (*p*NPP) as described previously.^31^ Consistent with the results of others,^48^ a positive signal at approximately 232 nm is observed in the far-UV region of the CD spectrum, which is characteristic of through-space interactions between Tyr26 and Trp66 forming an exciton in the native core of PagP.^48^ This band was observed in all CD spectra of 5 μM refolded PagP, obtained in the presence of 7 M urea and at an LPR of 3200:1, irrespective of the membrane composition (Fig. 3b). Additionally, the negative maximum observed in the far-UV CD spectra between 215 and 220 nm indicated that all the proteins had adopted the β-sheet structure of the native protein (Fig. 3b).^31,48^ By contrast, a spectrum of the protein taken in 7 M urea in the absence of liposomes lacked both of these characteristic features of native PagP (Fig. 3b). Additional evidence that PagP can fold to its native conformation in membranes containing either PE or PS was provided by the Trp fluorescence emission spectra of 0.4 μM PagP taken following incubation in the presence or absence of liposomes in 7 M urea. In the absence of liposomes, unfolded PagP exhibited low intensity fluorescence with a maximum at 350 nm (Fig. 3c). In contrast, following incubation with liposomes with the compositions detailed above, at an LPR of 3200:1, the fluorescence spectra of PagP showed high fluorescence intensity with a λ_max_ ∼ 335 nm and a shoulder at 350 nm (Fig. 3c), characteristic of the native protein as reported previously.^31^

Proteins refolded in each of the membrane compositions described above exhibited an ability to convert *p*NPP to *p-*nitrophenol, confirming the attainment of the native β-barrel structure of PagP. Interestingly, the activity measured was dependent on the membrane composition (0.117 ± 0.011 and 0.021 ± 0.017 nmol min^− 1^ μM^− 1^ in the *di*C_12:0_PC membranes containing 40% w/w *di*C_12:0_PS and 10% w/w *di*C_12:0_PE, respectively, compared with 0.068 ± 0.011 and 0.004 ± 0.001 nmol min^− 1^ μM^− 1^ for membranes containing solely *di*C_12:0_PC and for unfolded PagP, respectively^31^^)^. Together, the data indicate that, in addition to its ability to fold into membranes consisting solely of 100% *di*C_12:0_PC, PagP can also fold to a native conformation in membranes containing up to 10% w/w *di*C_12:0_PE or 40% w/w *di*C_12:0_PS.

### Phosphatidylethanolamine, but not phosphatidylserine, headgroups control a shift between alternative folding pathways

Having established that PagP can insert into membranes of differing phospholipid composition to yield a correctly folded, functional state, we next investigated the kinetics of PagP folding in 7 M urea using 0.4 μM PagP and *di*C_12:0_PC membranes containing 5–15% w/w *di*C_12:0_PE or 2.5–40% w/w *di*C_12:0_PS-lipids, at an LPR of 3200:1. In contrast to PagP folding at this concentration into pure *di*C_12:0_PC-bilayers, in which a single exponential phase followed the burst phase (Table 1), introducing 5% w/w *di*C_12:0_PE into the liposome bilayer resulted in double exponential kinetics (Table 2). Moreover, the data also suggested that the population of PagP molecules folding *via* the faster folding pathway, associated with *k*_f1_, decreased upon increasing the *di*C_12:0_PE-content such that the population folding with a slower rate constant, *k*_f2_, became dominant, concomitant with a decrease in burst phase amplitude, until folding occurred entirely following the slower pathway, associated with *k*_f2_, at 10% w/w *di*C_12:0_PE (Table 2). Increasing the *di*C_12:0_PE-content of the liposomes to 15% w/w resulted in very slow folding rates that could not be measured reliably (Table 2). In contrast with the results obtained with bilayers containing *di*C_12:0_PE, in all cases where *di*C_12:0_PS was present in the lipid bilayer, folding kinetics were described by a burst phase and a single exponential phase with a rate constant that decreased 20-fold with increasing *di*C_12:0_PS concentration in the lipid bilayer from 2.5% to 40% w/w (Table 2).

Taken together, the data show that variation in the headgroup composition of the phospholipid bilayer has a significant effect on the folding of PagP, depending on the precise phospholipids present. While an increase in acyl chain packing in the bilayer (which results from the presence of the PE headgroups^46^^)^ induced a switch from the fast to the slow folding pathways, changing the net charge of the membrane (by introduction of PS head groups within diC_12:0_PC) affects the rate of folding into the membrane but retains single exponential kinetics.

### Unfolding kinetics are independent of protein and lipid concentrations

**Fig. 4:**
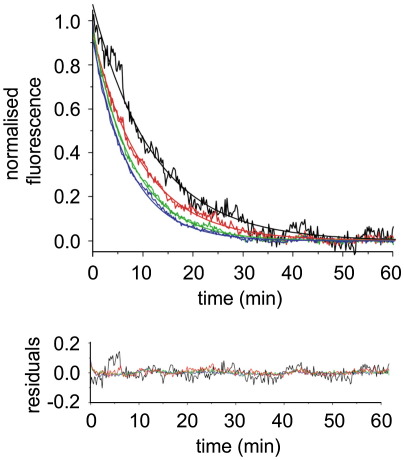
Top: unfolding kinetics of 0.05 (black), 0.1 (red), 0.2 (green) and 0.4 (blue) μM PagP in *di*C_12:0_PC liposomes at an LPR of 3200:1 in 10 M urea. Lines through the experimental data represent fits to a single exponential function. Bottom: residuals to single exponential fits. All experiments were performed in 50 mM sodium phosphate buffer, pH 8, at 25 °C.

To investigate whether the unfolding kinetics of PagP are also affected by the LPR, the PagP concentration or the phospholipid employed, we measured the unfolding kinetics of PagP in the presence of 10 M urea^31^ in *di*C_12:0_PC liposomes at different concentrations of PagP or at different LPRs (Table 3) or in liposomes containing different ratios of *di*C_12:0_PC and *di*C_12:0_PE or *di*C_12:0_PS (Table 4). Interestingly, under all conditions investigated, unfolding traces monitored by Trp fluorescence fitted well to single exponentials (Fig. 4 and Tables 3 and 4). In agreement with previous results,^30^ with the exception of the lowest PagP concentration (0.05 μM) and lowest LPR (400:1) investigated, the unfolding rate constants measured were largely not affected by either the protein concentration or LPR (Table 3). Inclusion of 20% or more *di*C_12:0_PS into *di*C_12:0_PC membranes had a modest effect and decreased the unfolding rates approximately 2-fold, while inclusion of *di*C_12:0_PE strongly reduced the unfolding rates by up to 14-fold (Table 4).

## Discussion

The intimate relationship between membrane proteins and the lipid bilayer is an inherent part of the membrane protein folding problem. A complete understanding of the mechanism of membrane protein folding, therefore, requires not only the delineation of the contributions of the amino acid sequence to the folding process,^30,49,50^ but also rationalization of the contributions of the physicochemical properties of the lipid bilayer.

### PagP folds through parallel pathways

**Fig. 5:**
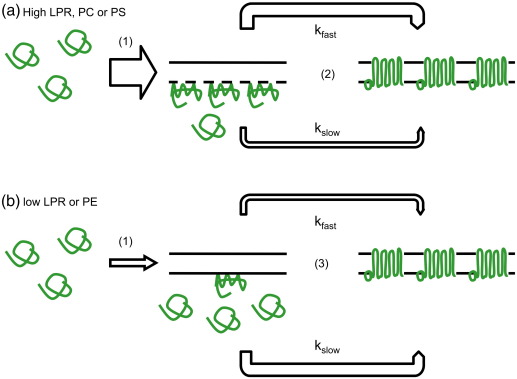
Schematic representation of the folding mechanism of PagP. Folding is initiated by rapid adsorption to the membrane (1). The amplitude of this process is increased with increasing LPRs in (a) and decreased by decreasing LPR or by incorporation of *di*C_12:0_PE in (b). Folding proceeds predominantly by a fast (2) or a slow (3) folding route, modulated by the bilayer properties determined by the LPR and *di*C_12:0_PE fraction in the liposome as indicated. The dotted line in (a) represents higher flexibility in the membrane compared to (b). Including *di*C_12:0_PS in the liposome does not change the preferred pathway but does influence the rate of insertion. The thickness of the arrows indicates the amplitude of the phases.

Investigations of the folding of monomeric β-barrel outer membrane proteins into lipid vesicles *in vitro* have often revealed complex, multiphase kinetics.^30,32,35,39^ In the cases of OmpA and OmpF, such multiple phases were suggested to arise from distinct intermediates along the path to the native state.^39,51^ By contrast, because of the absence of an experimentally detectable intermediate species, FomA was suggested to fold *via* parallel folding pathways postulated to arise from two distinct unfolded conformations (proximal and distal to the lipid surface).^35^ Using interrupted folding experiments of the type frequently used in the folding analysis of water-soluble proteins,^52–54^ we have directly demonstrated the existence of parallel folding pathways for PagP and its dependence on variations in the LPR at which folding is performed (Fig. 5): the slower of two pathways is outcompeted with increasing LPR to such an extent that, at sufficiently high LPR, the complete PagP population folds solely using the more rapid pathway.

We have previously reported single exponential refolding transients of PagP into diC_12:0_PC liposomes at an LPR of 3200:1 wherein PagP refolds from a liposome-adsorbed state.^30^ Although in the previous study, folding rate constants were only determined for urea concentrations ≥ 7.8 M, the observed linear dependence of the logarithm of the rate constants on the denaturant concentration^30^ allows the rate constant at 7 M urea to be estimated by extrapolation. Interestingly, an estimated rate constant of 0.51 min^− 1^ (± 12%, resulting from the confidence of the fit) correlates well with the rate constant *k*_f1_ measured here under identical physicochemical conditions, but with the folding reaction initiated from a completely unfolded and lipid dissociated state. We have previously shown that the large burst phase preceding the phase resolved by a single exponential in the latter reaction represents rapid association of PagP with the membrane surface.^30^ Together, the data suggest, therefore, that membrane association in the burst phase directly precedes folding *via* the rapid pathway to the native state at an LPR of 3200:1 and a PagP concentration of 0.4 μM. What then is the mechanism of the folding pathway associated with the slower rate constant?

Since unfolding transients could consistently be fitted to single exponential functions under a variety of conditions, there is a lack of evidence supporting the existence of an alternatively folded native-like conformation of PagP that could, in principle, give rise to the slower folding pathway. Moreover, interrupted refolding experiments also failed to reveal alternative or partially folded states. In the case of FomA folding, Pocanschi *et al.* hypothesized that temporary saturation of the membrane surface at low LPR by a fraction of the protein molecules establishes a second protein population that remains folding competent in solution, poised to adsorb onto the membrane upon the exposure of free lipid surface.^35^ The significant decrease in burst phase amplitude as a function of decreasing LPR during the folding of PagP is consistent with this idea. An additional factor contributing to the observed decrease in folding (and unfolding) rate constants as a function of either LPR or protein concentration could result from an increase in the bending modulus of the membrane, a measure for the decrease in membrane flexibility.^22,23^ This, for example, occurs due to bilayer deformation induced by the inclusion of two or more protein molecules in the presence of a hydrophobic mismatch. Although some theoretical descriptions exist,^23,55^ experimental verification of such deformations and their propagation through the membrane are not straightforward as such effects are protein specific.^29,56^ The decrease in folding and unfolding rate constants of PagP at an LPR of 400:1, nonetheless, is in agreement with an increase in bending modulus.

### Properties of lipid bilayers that contribute to the folding mechanism of PagP

The effect of membrane flexibility on the (un)folding rates of PagP into *di*C_12:0_PC liposomes is clearly demonstrated here by the inclusion of lipids known to modulate the membrane curvature stress and bilayer stiffness. For example, inclusion at high LPR (3200:1) of *di*C_12:0_PE^57,58^ was found to result in a reduction in the burst phase amplitude and a switch to the slower folding pathway (Fig. 5), as also observed at lower LPRs in pure *di*C_12:0_PC liposomes. Reduced rate constants in *di*C_12:0_PE-containing liposomes were also found in unfolding traces of proteins embedded into membranes containing *di*C_12:0_PE. We suggest, therefore, that membrane deformation, possibly involving thinning of the hydrophobic core, upon contact with the unfolded protein chain may provide an active driving force for membrane protein folding and assist in the insertion process. In contrast to *di*C_12:0_PE, *di*C_12:0_PS-lipids do not change bilayer stiffness of *di*C_12:0_PC-membranes^45,59,60^ and, consequently, did not result in a change in flux into the slow folding pathway when included in a *di*C_12:0_PC-bilayer. While the role of surface electrostatics in reducing (un)folding rates is more difficult to rationalize, in part because of the lack of high-resolution structures of the native and unfolded states of the protein in the presence of membranes, increased repulsion between the charges on the PagP molecules (p*I* ∼ 5.8) and the negatively charged membrane surface potentially provides an explanation for the decreased folding rates into such lipid mixtures.

The modulating capacity of the lipid membrane on the folding kinetics of PagP and many other β-barrel membrane proteins *in vitro*^26,35,40,61^ suggests that intrinsic properties of the membrane help to guide nonnative outer membrane proteins towards their native structure. Although the mechanism of membrane insertion is not yet understood in the complex lipopolysaccharide environment of the bacterial outer membrane, which is known to be rather rigid,^62^ the assembly of β-barrel membrane proteins is known to be assisted by molecular chaperones *in vivo*.^63,64^ It would therefore be interesting to investigate how bilayer properties affect the action of chaperones to assist in rapid insertion of refolding PagP in vitro or *vice versa* to cast some light on how Nature might balance these effects to create a folding-competent environment.

## Materials and Methods

### Protein purification

PagP was expressed in *E. coli* strain Rosetta 2 (Novagen), and bacteria were grown in LB medium. The produced protein was purified from inclusion bodies under denaturing conditions as described previously.^42^ Typically, 50 mg of purified protein was obtained per liter of culture and stored at − 20 °C either as a pellet precipitated from the denaturing buffer by dialysis against distilled water or as a solution in 6 M guanidine hydrochloride (Gdn-HCl), with a typical protein concentration of 0.5 mM.

### Preparation of liposomes

Appropriate mixtures of lipids (*di*C_12:0_PC, *di*C_12:0_PE, *di*C_12:0_PS; Avanti, Alabaster, AL, USA) dissolved in a 9:1 chloroform–methanol mixture were dried on the bottom of a test tube under a gentle stream of nitrogen gas and then in a desiccator under high vacuum. The resulting thin lipid films were hydrated to give a 40 mM lipid solution in 50 mM sodium phosphate buffer (pH 8). Vesicles thus formed were extruded 11 times through 100 nm-pore-size polycarbonate membranes (Nucleopore, Whatman, Clifton, NJ) using a mini-extruder (Avanti).

### Folding and unfolding kinetics of PagP in preformed liposomes

Refolding of PagP was initiated by mixing 0.4 μM PagP, denatured in 6 M Gdn-HCl, with lipid vesicles at the lipid-to-protein molar ratios indicated in the text in the presence of 7 M urea in 50 mM sodium phosphate buffer (pH 8) at 25 °C, typically diluting the Gdn-HCl-containing solution approximately 1000-fold. Refolding kinetics were monitored by following fluorescence emission from endogenous Trp residues at 335 nm upon excitation at 280 nm, using a slit width of 3 mm and a cuvette of 10 mm path length in a Photon Technology International (PTI) fluorimeter equipped with a 4-cell changer (Ford, UK). For the measurement of unfolding kinetics, PagP was first allowed to refold in liposomes as described above, after which the denaturant concentration was increased to 10 M urea, while maintaining all other experimental conditions the same. In all cases, the traces were corrected for photobleaching by subtracting a linear function. The data were then fitted to $y=A\prime_{\text{burst}}+A\prime_{\text{1}}(1-e^{-k_{1}t})+A\prime_{\text{2}}(1-e^{-k_{2}t})$. The fluorescence signal of the unfolded state in 7 M urea was used as a baseline to determine the burst amplitude. To assess the contribution of the amplitudes of the slow exponential phases (*A*_1_′ + *A*_2_′) to the overall folding reaction, we calculated the percentage of these slow phase amplitudes relative to the total amplitude using % *A*_total_ = (*A*_1_′ + *A*_2_′/*A*_1_′ + *A*_2_′ + *A*_burst_′) × 100. To quantify how the membrane and protein properties affected the relative amplitudes of each slow exponential phase, we normalized folding trajectories between 0 and 1 after subtraction of the burst phase amplitude (*A*_burst_′). Data were then fitted using $y=A_{\text{0}}+A_{\text{1}}(1-e^{-k_{1}t})+A_{\text{2}}(1-e^{-k_{2}t})$. Here, *A*_0_ is an additional offset to account for over or underestimation of *A*_burst_′, leading to an error in the determination of (*A*_1_ + *A*_2_) of less than 5% in most cases. The error was 7% in the following cases: (a) at an LPR of 1600:1 with 0.75 μM PagP and (b) at an LPR of 3200:1 in the presence of 2.5% and 40% *di*C_12:0_PS in *di*C_12:0_PC-liposomes. The confidence error was 12% at an LPR of 2400:1 with 0.05 μM PagP. Unfolding transients were fitted using $y=A_{\text{0}}+A_{\text{1}}e^{-k_{u}t}$. Curve fitting errors are reported for all kinetic rate constants.

### Interrupted folding experiments

Folding was initiated at a urea concentration of 7 M by mixing 0.4 μM PagP with *di*C_12:0_PC liposomes at an LPR of 400:1 in 50 mM sodium phosphate buffer, pH 8, 25 °C. After a time delay, *t*_*i*_, a 500 μl sample was taken and mixed with 1 ml of 11 M urea in 50 mM sodium phosphate buffer, pH 8. The subsequent unfolding signal was followed by discontinuous measurements over 4–5 h at 335 nm upon excitation at 280 nm with excitation and emission slit widths of 3 nm and a path length of 10 mm, using a PTI fluorimeter equipped with a thermally controlled 4-cell changer. The temperature was held at 25 °C using a water bath. The resultant trace was fitted to a single exponential in Origin Pro v. 7.5 using the equation *y* = *A*_0_ + *A*_1_*e*^− *kt*^. The amplitudes, *A*, were normalized by dividing each amplitude at *t*_*i*_ by the unfolding amplitude of the completely refolded protein (at *t*_*i*_ = 90 min) and fitted to a single or double exponential function.

### Spectroscopy

Trp fluorescence emission spectra of 0.4 μM PagP were obtained between 300 and 380 nm at 25 °C and a slit width of 3 mm using an excitation wavelength of 280 nm and a path length of 10 mm in a PTI fluorimeter. CD spectra of 5 μM PagP in liposomes were taken on a Jasco 715 spectropolarimeter between 200 and 250 nm using a cell with 1 mm path length, a scan speed of 50 nm min^− 1^ and a bandwidth of 1 nm. The temperature was held at 25 °C using a Jasco PTC-351S peltier system.

### Activity assays

The enzymatic assay for PagP activity after refolding into lipid vesicles was performed as described previously.^14^ Briefly, *p*NPP was added to a liposome solution, after which vesicles were sonicated to obtain a dispersion of liposomes and *p*NPP. PagP was added in the presence of 7 M urea as described above. Substrate conversion was followed at 410 nm for 20 min.

## Figures and Tables

**Table 1:** Fitting parameters of wild-type PagP folding into 100 nm 100% *di*C_12:0_PC-liposomes in 7 M urea (in 50 mM sodium phosphate buffer, pH 8, 25 °C)

LPR	[PagP] (μM)	*k*_f1_ (min^− 1^)	*k*_f2_ (min^− 1^)	*A*_1_	*A*_2_	% *A*_total_^a^
3200:1	0.05	0.22 ± 0.01	NA	0.98 ± 0.01	NA	56
3200:1	0.1	0.43 ± 0.01	NA	0.97 ± 0.01	NA	48
3200:1	0.2	0.59 ± 0.01	NA	1.02 ± 0.01	NA	47
3200:1	0.4	0.56 ± 0.01	NA	1.01 ± 0.01	NA	55
2400:1	0.05	0.21 ± 0.02	0.05 ± 0.01	0.66 ± 0.05	0.55 ± 0.05	29
2400:1	0.1	0.32 ± 0.01	NA	1.00 ± 0.01	NA	34
2400:1	0.2	0.53 ± 0.01	NA	1.06 ± 0.01	NA	37
2400:1	0.4	0.66 ± 0.02	NA	1.05 ± 0.02	NA	34
1600:1	0.05	NA	0.08 ± 0.00	NA	0.99 ± 0.01	42
1600:1	0.075	NA	0.06 ± 0.00	NA	1.01 ± 0.00	48
1600:1	0.1	0.38 ± 0.02	0.11 ± 0.00	0.48 ± 0.03	0.55 ± 0.03	48
1600:1	0.2	0.33 ± 0.01	NA	0.96 ± 0.01	NA	49
1600:1	0.4	0.52 ± 0.01	NA	0.99 ± 0.01	NA	57
800:1	0.05	0.35 ± 0.07	0.06 ± 0.00	0.09 ± 0.01	0.98 ± 0.01	65
800:1	0.15	0.20 ± 0.02	0.04 ± 0.00	0.24 ± 0.01	0.78 ± 0.01	70
800:1	0.2	0.39 ± 0.04	0.12 ± 0.01	0.35 ± 0.04	0.66 ± 0.04	76
800:1	0.4	0.34 ± 0.00	NA	0.97 ± 0.01	NA	76
400:1	0.2	0.21 ± 0.01	0.06 ± 0.00	0.51 ± 0.02	0.49 ± 0.02	88
400:1	0.3	0.58 ± 0.04	0.05 ± 0.00	0.17 ± 0.01	0.85 ± 0.00	88
400:1	0.4	0.11 ± 0.01	0.02 ± 0.00	0.34 ± 0.01	0.67 ± 0.01	85

NA, not applicable because a good fit was obtained to a single exponential.

a % *A*_total_ is the percentage contribution of the slow phase(s) to the total amplitude (burst phase amplitude + slow phase amplitudes; see Materials and Methods for details).

**Table 2:** Fitting parameters of wild-type PagP folding in liposomes with varying composition under the various conditions studied in the presence of 7 M urea (in 50 mM sodium phosphate buffer, pH 8, 25 °C)

Liposome composition	LPR	[PagP] (μM)	*k*_f1_ (min^− 1^)	*k*_f2_ (min^− 1^)	*A*_1_	*A*_2_	% *A*_total_^a^
*di*C_12:0_PC with							
5% *di*C_12:0_PE	3200:1	0.4	0.16 ± 0.01	0.04 ± 0.01	0.43 ± 0.01	0.58 ± 0.02	64
10% *di*C_12:0_PE	3200:1	0.4	NA	0.02 ± 0.00	NA	1.00 ± 0.00	78
15% *di*C_12:0_PE	3200:1	0.4	NA	0.00 ± 0.00	NA	ND	90
*di*C_12:0_PC with							
2.5% *di*C_12:0_PS	3200:1	0.4	0.19 ± 0.02	NA	1.01 ± 0.01	NA	63
5% *di*C_12:0_PS	3200:1	0.4	0.20 ± 0.00	NA	1.01 ± 0.01	NA	64
10% *di*C_12:0_PS	3200:1	0.4	0.11 ± 0.00	NA	1.01 ± 0.01	NA	74
20% *di*C_12:0_PS	3200:1	0.4	0.03 ± 0.00	NA	1.05 ± 0.00	NA	74
30% *di*C_12:0_PS	3200:1	0.4	0.02 ± 0.00	NA	1.03 ± 0.01	NA	62
40% *di*C_12:0_PS	3200:1	0.4	0.01 ± 0.00	NA	1.08 ± 0.02	NA	63

NA, not applicable because a good fit was obtained to a single exponential. ND, not determined owing to the extremely slow folding rate under these conditions.

a % *A*_total_ is the percentage contribution of the slow phase(s) to the total amplitude (burst phase amplitude + slow phase amplitudes); see Materials and Methods for details).

**Table 3:** Fitting parameters of wild-type PagP unfolding from 100% *di*C_12:0_PC liposomes under the various conditions studied in the presence of 10 M urea (in 50 mM sodium phosphate buffer, pH 8, 25 °C)

LPR	[PagP] (μM)	*k*_u_ (min^− 1^)	*A*_1_
3200:1	0.05	0.08 ± 0.00	1.08 ± 0.01
3200:1	0.1	0.10 ± 0.00	0.95 ± 0.01
3200:1	0.2	0.13 ± 0.00	0.96 ± 0.00
3200:1	0.4	0.14 ± 0.00	0.92 ± 0.00
2400:1	0.05	0.04 ± 0.00	0.97 ± 0.00
2400:1	0.1	0.10 ± 0.00	0.99 ± 0.01
2400:1	0.2	0.12 ± 0.00	0.95 ± 0.00
2400:1	0.4	0.13 ± 0.00	0.90 ± 0.00
1600:1	0.05	0.08 ± 0.00	0.99 ± 0.01
1600:1	0.1	0.09 ± 0.00	0.99 ± 0.01
1600:1	0.2	0.12 ± 0.00	1.01 ± 0.00
1600:1	0.4	0.13 ± 0.00	0.96 ± 0.00
800:1	0.1	0.09 ± 0.00	0.98 ± 0.01
800:1	0.2	0.09 ± 0.00	0.98 ± 0.00
800:1	0.4	0.10 ± 0.00	0.94 ± 0.00
400:1	0.1	0.01 ± 0.00	0.96 ± 0.03
400:1	0.2	0.03 ± 0.00	0.86 ± 0.00
400:1	0.4	0.05 ± 0.00	0.90 ± 0.00

Fitting equation: *nf* = *A*_0_ + *A*_1_exp(− *k*_u_*t*), in which *nf* = normalized fluorescence and *t* = time.

**Table 4:** Fitting parameters of wild-type PagP unfolding in liposomes with varying composition under the various conditions studied in the presence of 10 M urea (in 50 mM sodium phosphate buffer, pH 8, 25 °C)

Liposome composition	LPR	[PagP] (μM)	*k*_u_ (min^− 1^)	*A*_1_
*di*C_12:0_PC with				
0% *di*C_12:0_PE^a^	3200:1	0.4	0.14 ± 0.00	0.92 ± 0.00
5% *di*C_12:0_PE	3200:1	0.4	0.04 ± 0.00	0.93 ± 0.00
10% *di*C_12:0_PE	3200:1	0.4	0.01 ± 0.00	0.92 ± 0.00
15% *di*C_12:0_PE	3200:1	0.4	0.01 ± 0.00	0.95 ± 0.00
*di*C_12:0_PC with				
0% *di*C_12:__0_PS^a^	3200:1	0.4	0.14 ± 0.00	0.92 ± 0.00
2.5% *di*C_12:0_PS	3200:1	0.4	0.13 ± 0.00	0.92 ± 0.01
5% *di*C_12:0_PS	3200:1	0.4	0.22 ± 0.00	1.05 ± 0.00
10% *di*C_12:0_PS	3200:1	0.4	0.11 ± 0.00	1.06 ± 0.00
20% *di*C_12:0_PS	3200:1	0.4	0.06 ± 0.00	1.06 ± 0.00
30% *di*C_12:0_PS	3200:1	0.4	0.08 ± 0.00	1.01 ± 0.00
40% *di*C_12:0_PS	3200:1	0.4	0.08 ± 0.00	1.00 ± 0.00

a Data taken from Table 3.
